# Social Media Listening to Understand the Lived Experience of Presbyopia: Systematic Search and Content Analysis Study

**DOI:** 10.2196/18306

**Published:** 2020-09-21

**Authors:** James S Wolffsohn, Claudia Leteneux-Pantais, Sima Chiva-Razavi, Sarah Bentley, Chloe Johnson, Amy Findley, Chloe Tolley, Rob Arbuckle, Jyothi Kommineni, Nishith Tyagi

**Affiliations:** 1 Optometry and Vision Science College of Health and Life Sciences Aston University Birmingham United Kingdom; 2 Novartis Pharma AG Basel Switzerland; 3 Patient-Centered Outcomes Adelphi Values Ltd Bollington United Kingdom; 4 Novartis Business Services Product Lifecycle Services Hyderabad India

**Keywords:** presbyopia, near vision, social media, social media listening, infodemiology

## Abstract

**Background:**

Presbyopia is defined as the age-related deterioration of near vision over time which is experienced in over 80% of people aged 40 years or older. Individuals with presbyopia have difficulty with tasks that rely on near vision. It is not currently possible to stop or reverse the aging process that causes presbyopia; generally, it is corrected with glasses, contact lenses, surgery, or the use of a magnifying glass.

**Objective:**

This study aimed to explore how individuals used social media to describe their experience of presbyopia with regard to the symptoms experienced and the impacts of presbyopia on their quality of life.

**Methods:**

Social media sources including Twitter, forums, blogs, and news outlets were searched using a predefined search string relating to symptoms and impacts of presbyopia. The data that were downloaded, based on the keywords, underwent manual review to identify relevant data points. Relevant posts were further manually analyzed through a process of data tagging, categorization, and clustering. Key themes relating to symptoms, impacts, treatment, and lived experiences were identified.

**Results:**

A total of 4456 social media posts related to presbyopia were identified between May 2017 and August 2017. Using a random sampling methodology, we selected 2229 (50.0%) posts for manual review, with 1470 (65.9%) of these 2229 posts identified as relevant to the study objectives. Twitter was the most commonly used channel for discussions on presbyopia compared to forums and blogs. The majority of relevant posts originated in Spain (559/1470, 38.0%) and the United States (426/1470, 29.0%). Of the relevant posts, 270/1470 (18.4%) were categorized as posts written by individuals who have presbyopia, of which 37 of the 270 posts (13.7%) discussed symptoms. On social media, individuals with presbyopia most frequently reported experiencing difficulty reading small print (24/37, 64.9%), difficulty focusing on near objects (15/37, 40.5%), eye strain (12/37, 32.4%), headaches (9/37, 24.3%), and blurred vision (8/37, 21.6%). 81 of the 270 posts (30.0%) discussed impacts of presbyopia—emotional burden (57/81, 70.4%), functional or daily living impacts (46/81, 56.8%), such as difficulty reading (46/81, 56.8%) and using electronic devices (21/81, 25.9%), and impacts on work (3/81, 3.7%).

**Conclusions:**

Findings from this social media listening study provided insight into how people with presbyopia discuss their condition online and highlight the impact of presbyopia on individuals’ quality of life. The social media listening methodology can be used to generate insights into the lived experience of a condition, but it is recommended that this research be combined with prospective qualitative research for added rigor and for confirmation of the relevance of the findings.

## Introduction

Presbyopia is the most common physiological change occurring in the adult eye. In presbyopia, the elasticity of the lens begins to deteriorate, causing universal near vision impairment with increasing age [1,2]. It is estimated that presbyopia is experienced in over 80% of people aged 40 years or above in western countries [1]. There is currently no way to stop or reverse the normal aging process that causes presbyopia; near vision impairments associated with presbyopia are typically corrected with glasses, contact lenses, surgery, or the use of a magnifying glass [3]. In 2015, it was estimated that presbyopia affected approximately 1.8 billion people (25% of the world population), with 826 million experiencing near vision impairments because they had no (or inadequate) vision correction [4]. As a result, the global unmet need for presbyopia correction methods in 2015 was estimated to be 45% [4].

Presbyopia impacts many domains of quality of life including difficulty with near vision tasks, such as reading printed text, using a smartphone, or threading a needle [5,6]. Difficulty performing these tasks can worsen over time and is typically more significant if the lighting is not optimal [2,6]. Such problems can, in turn, impact work productivity, social interactions, household activities, and emotional well-being [7,8]. Emotional impacts associated with presbyopia include having to rely more on others, feeling ashamed, and feeling embarrassed due to poor vision [8]. Individuals who do not wear glasses or contact lenses may experience headaches and eye strain due to their difficulty focusing on objects [9]. Some individuals with presbyopia describe holding objects (eg, a menu) progressively farther away from their eyes in order to be able to focus on them [1].

Minimal qualitative research [6] into the lived experience of presbyopia (not limited to a single form of correction) has been published, and what has been published focuses generally on refractive errors rather than being specific to presbyopia. Traditionally qualitative research is conducted via interviews or focus groups. However, social media sources can now be utilized to provide qualitative data for a large sample across multiple countries [10]. Approximately 68% of all US adults use Facebook, while over 20% of US adults use Instagram, Pinterest, LinkedIn, and Twitter [11]. Peer-to-peer exchange of health information is popular online; a recent study found that 51% of Americans had used social networking, and a further 66% had looked at blogs for health information [12].

Social media reviews are increasingly being used to investigate the patient experience of health conditions such as dry eye and chronic obstructive pulmonary disease [13-15]. Other studies have used social media listening to investigate the emotional impact of caregivers who look after patients with leukemia [16] and to identify posts discussing potential misuse or nonmedical use of antidepressants [17]. One study found social media listening generated more concepts relevant to the lived experience of specific conditions in comparison to those generated by concept elicitation interviews and the methodology of group concept mapping [18]. It has been theorized that some individuals may feel more comfortable discussing socially embarrassing symptoms online than discussing them in an interview or focus group setting [18,19]. It is acknowledged that the depth of data collected by social media reviews can be limited in comparison to those collected by methods such as concept elicitation interviews, particularly in cases where character counts are limited for each post (eg, Twitter with a 140 character limit per post, at the time of this research) [18]. To the authors' knowledge, there is currently no published research exploring how presbyopia is discussed on social media; this study aims to address this gap.

This study aimed to explore how individuals with presbyopia use social media to describe their experiences. Specifically, the study explored how the social media population described the visual and nonvisual symptoms that they experience as a result of presbyopia and the impacts that presbyopia has on their health-related quality of life (HRQoL). A secondary objective was to explore individuals’ experiences of diagnosis and treatment or vision correction method options.

## Methods

### Study Design

This study was a noninterventional retrospective analysis of social media data available in the public domain.

### Search Strategy

A predefined search string was used to identify social media posts and discussions that were relevant to the lived experience of presbyopia. The search string terms were initially identified through a literature search and review of online patient forums. Two approaches were taken to develop the final search string. The first approach was to search social media sources for indication-related keywords only (in English and translated to local languages). This first approach helped identify any further associated symptom or impact terms. The second approach involved searching social media sources using a combination of indication-related keywords and other disease journey–related keywords such as *symptoms*, *diagnosis*, and *vision correction*. Based on the results obtained from these two preliminary searches, appropriate terms were included in the search strategy.

The resulting search string (Multimedia Appendix 1) contained terminology related to the symptoms experienced, impacts of presbyopia, and vision correction options. Three key generic presbyopia search terms (*presbyopia*, *long sightedness*, and *elasticity*) were also translated into five additional languages (German, French, Spanish, Italian, and Japanese) and included in the search query. Boolean operators (AND, OR) were used to combine the keywords into a single search string.

### Data Collection

Sales Force Social Studio database [20] was used to conduct the searches. The search terms (listed in Multimedia Appendix 1) were inputted into the database, and the Sales Force Social Studio software identified posts that matched the search terms across the following media channels: Twitter, forums, blogs, and news posts. Relevant forums and blog posts were identified on online community websites and discussion boards such as Medhelp and Optiboard. News posts were identified from general news websites across different countries.

Social media posts were searched in and identified from the United States, France, Germany, Italy, Spain, the United Kingdom, and Japan. All relevant posts were downloaded, and a random sampling methodology was employed using a simple randomization technique to reduce the number of posts by 50%. The number of posts were reduced by 50% to ensure it was a manageable amount for the research team to manually review. Posts that were originally non-English were translated into to English using Google translate. Posts were then manually reviewed by researchers against predefined criteria (Multimedia Appendix 2) to ensure they were relevant to the study objectives.

### Data Analysis

Relevant posts were automatically tagged by channel type (eg, Twitter, Forums, Blogs, or News) and, where possible, manually categorized by type of stakeholder (eg, individuals with presbyopia, physicians, researchers, clinics, support groups, company handles, and media), sentiment (positive, neutral, or negative), key themes of discussion, and lived experience. The stakeholder category was established through the grammatical tense of the post. For example, a post that discussed presbyopia in the first person such as “I have difficulty reading because of my presbyopia” was categorized as an individual with presbyopia. Due to the amount of data evaluated, it was not possible to review every profile to confirm stakeholder categorization, and as expected, for many posts that were reviewed, the stakeholder category could not be deduced based on the post content. However, these findings give an indication of the approximate proportions of stakeholders discussing presbyopia online.

State-of-mind analysis was used to explore how individuals felt about presbyopia [21]. State-of-mind analysis is an analyst interpretation of the verbatim and focused on the key psychological and emotional state of audience. In order to maintain the consistency of the interpretation, the analysis went through three levels of review (review from analyst, quality control manager, and project manager).

To illustrate and exemplify the results reported, the authors have included a number of quotations from the social media sources. The authors took several steps to anonymize these publicly reported quotations to ensure direct quotations from posts cannot be traced online. First, any direct quotations that were originally non-English were translated and as a result are not identifiable. Second, the username of the post’s author was removed. Third, any individual patient or caregiver information was anonymized. Finally, any originally English quotations were edited to ensure these cannot be identified online. To edit quotations, the sentence structure and certain words were replaced by synonyms. This was done in a way that ensured the meaning of the quotation was retained. The edited quotations were reviewed by the research team, and consensus was reached.

## Results

### Overview of Data

**Figure 1:**
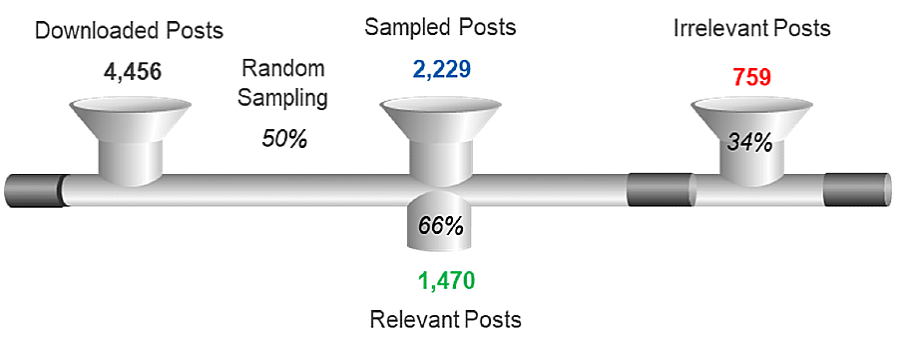
Process of analysis of post relevancy.

Social media posts were collated from May 2017 to August 2017. Figure 1 shows the data relevancy process that was conducted to identify the most relevant social media posts. A total of 4456 social media posts were obtained from the initial search. The random sampling methodology selected 2229 (50.0%) posts for manual review, with 1470 (65.9%) of these 2229 posts identified as relevant to the study objectives.

Twitter emerged as the most commonly used social media channel (Multimedia Appendix 3) and was the source of the majority of relevant posts (1182/1470, 80.4%), compared with the amount of posts derived from forums (164/1470, 11.2%), blogs (109/1470, 7.4%), and news sources (15/1470, 1.0%). Around 62.0% (733/1182) of discussions on Twitter were focused on disease awareness with symptoms of presbyopia being one of the most prominently tweeted topics. Forums contained the highest level of conversation among the social media population. Discussions on forums were predominantly about quality of life (60/164, 36.6%) and treatment or correction methods (98/164, 59.8%). Discussions on blogs were primarily centered on lifestyle (23/109, 21.1%) and treatment or correction method options (66/109, 60.6%).

The country of origin of the relevant posts was analyzed (Multimedia Appendix 4). The majority of posts originated in Spain (559/1470, 38.0%) or the United States (426/1470, 29.0%). Fewer posts originated from France (147/1470, 10.0%), the United Kingdom (118/1470, 8.0%), Japan (103/1470, 7.1%), Italy (88/1470, 6.0%), and Germany (15/1470, 1.0%).

**Figure 2:**
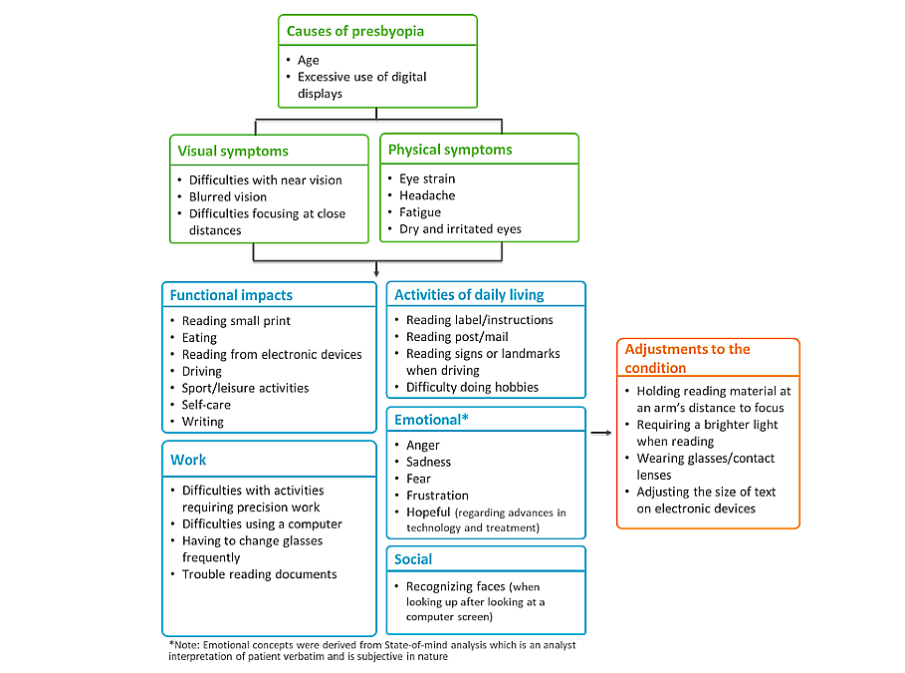
Conceptual model of social media listening findings.

A total of 270 posts (270/1470, 18.4%) were categorized as posts from individuals who had presbyopia based on the language used (eg, “I am diagnosed”; “I have this condition”). Based on limited data (27 discussions), most of the individuals (19/27, 70.4%) were aged between 40 and 70 years, as would be expected given the typical age range of presbyopia. A peak in social media discussions was observed from May 29, 2017 to June 19, 2017, due to a number of tweets about presbyopia awareness in Spain, the United States, and Japan. In the week of July 31, 2017 to August 7, 2017, a progressive lens was launched and led to an increase in tweets from individuals with presbyopia and health care professionals. A conceptual model (see Figure 2) was formulated to provide an overview of the key causes, symptoms, and impacts that were reported by the presbyopia population on social media. The model also summarizes the key adjustments described to help individuals with presbyopia better cope with the impacts of presbyopia.

### Symptoms of Presbyopia

Of the 270 posts categorized as having been posted by individuals with presbyopia (based on the language used), 37 posts referred to symptoms of presbyopia (37/270, 13.7%). The majority of data reported by presbyopic individuals relating to symptoms of presbyopia were obtained via Twitter (21/37, 56.8%), although some discussions were identified in forums (10/37, 27.0%) and blogs (5/37, 13.5%). The social media population appeared to be aware of their near vision being impaired and discussed symptoms such as difficulty reading small print (24/37, 64.9%), diminished ability to focus on near objects (15/37, 40.5%), eye strain (12/37, 32.4%), headaches (9/37, 24.3%), and blurred vision (8/37, 21.6%).

I have no difficulty reading The Washington Post but I have to strain to read print that is smaller in size.

### Impacts of Presbyopia

A total of 81 posts referred to impacts that presbyopia has on an individual’s life (81/270, 30.0%; see Table 1). The majority of data related to impacts of presbyopia were obtained via forums (42/81, 51.9%) and Twitter (30/81, 37.0%), and fewer were obtained from blogs (9/81, 11.1%). The social media population discussed functional or daily life impacts of presbyopia including difficulties reading (46/81, 56.8%), difficulty using digital devices (21/81, 25.9%), and limitations in sport and leisure activities (8/81, 9.9%). Individuals with presbyopia discussed a number of difficulties related to reading including reading text in a small font size (16/46, 34.8%), reading printed text in books (13/46, 28.3%), and reading at a distance when presbyopia becomes more severe (9/46, 19.6%).

**Table 1:** Impacts of presbyopia reported on social media.

Impact	Posts^a^ (n=81), n (%)
Emotional	57 (70.4)
Reading	46 (56.8)
Using digital devices	21 (25.9)
Sports and leisure activities	8 (9.9)
Work	3 (3.7)
Driving	2 (2.5
Recognizing people	2 (2.5)
Putting make-up on	1 (1.2)
Eating	1 (1.2)

^a^Posts could describe more than one.

I used to be able to read the comics from a distance. Actually, I can still do that. What I can't do is read them if they're right in front of me.

The letters “dance,” there are blurring even when looking from a distance, there is more visual fatigue and we become more dependent on a good light to be able to see the details up close. When the distance is not enough or is uncomfortable is when you start to use glasses of near vision.

The social media population also reported difficulties using digital devices (21/81, 25.9%), specifically mobile phones (11/21, 52.4%), televisions (4/21, 19.0%), computers (4/21, 19.0%), and laptops (3/21, 14.3%).

The worst of all is that I can not see “the mobile” without my presbyopic glasses.

Impacts of presbyopia relating to specific sports and leisure activities were discussed on social media (8/81, 9.8%) including difficulties diving (4/8, 50.0%), cycling (2/8, 25.0%), skiing (1/8, 12.5%), walking (1/8, 12.5%), and playing piano (1/8, 12.5%).

I find for walking that if I don't wear contacts I find it harder judging where the ground is on rough terrain, so the contacts help for that too.

Other impacts of activities of daily living reported by individuals on social media included driving (2/81, 2.5%), recognizing people (2/81, 2.5%), putting makeup on (1/81, 1.2%), and eating (1/81, 1.2%).

People may refer to it as “reading vision,” but it is the vision used for other near activities, such as eating, putting on make-up

In terms of work impacts, 3 posts (3/81, 3.7%) discussed difficulties using a computer (1/3, 33.3%), recognizing people (1/3, 33.3%), and using a needle (1/3, 33.3%).

Presbyopia affects an individual’s ability to enjoy and carry out a range of near vision activities – from reading, writing to precision tasks required in the workplace.

A total of 57 posts (57/81, 70.4%) relating to the emotional impact of presbyopia were identified in this analysis, with individuals with presbyopia typically reporting feelings of sadness (35/57, 61.4%), happiness due to a positive treatment experience, new reading technology, or not needing to depend on glasses (9/57, 15.8%), anger (7/57, 12.3%), and fear (6/57, 10.5%).

I have a little difficulty to tell the difference between the 3 and 8, and the 6 and 8 !!!! It's very annoying!!!

### Adjustments to Presbyopia

A number of posts discussed adjustments that individuals made to help them cope with the effects of presbyopia (154/270, 57.0%). These adjustments were primarily using glasses (87/154, 56.5%) or contact lenses (59/154, 38.3%). However, a few also referred to adjusting the size of text on an electronic device (4/154, 2.6%), holding reading material farther away than an arm’s length (2/154, 1.3%), and requiring a bright light (2/154, 1.3%).

I feel that my arms are now too short, and at the same time I find it hard to view from far, it could be presbyopia.

### Experience of Diagnosis of Presbyopia

A total of 51 posts (51/270, 18.9%) discussed diagnosis of presbyopia. Of these, 90.2% (46/51) reported that they were diagnosed by eye examination, and 9.8% (5/51) reported that they self-diagnosed their presbyopia. Out of those who reported they were diagnosed by eye examination, these included tests of visual acuity (14/46, 30.4%), retinal examination (13/46, 28.3%), slit lamp (12/46, 26.1%), a visual field test (11/46, 23.9%), evaluation of eye muscle integrity (11/46, 23.9%), refraction (2/46, 4.3%), and other tests (8/46, 17.4%).

It is the easiest way to determine presbyopia as a presbyopia based on whether you can read the letters of the newspaper from the position 30 cm away from the eyes or can not read.

Self diagnosis

### Experience of Vision Correction Methods for Presbyopia

**Figure 3:**
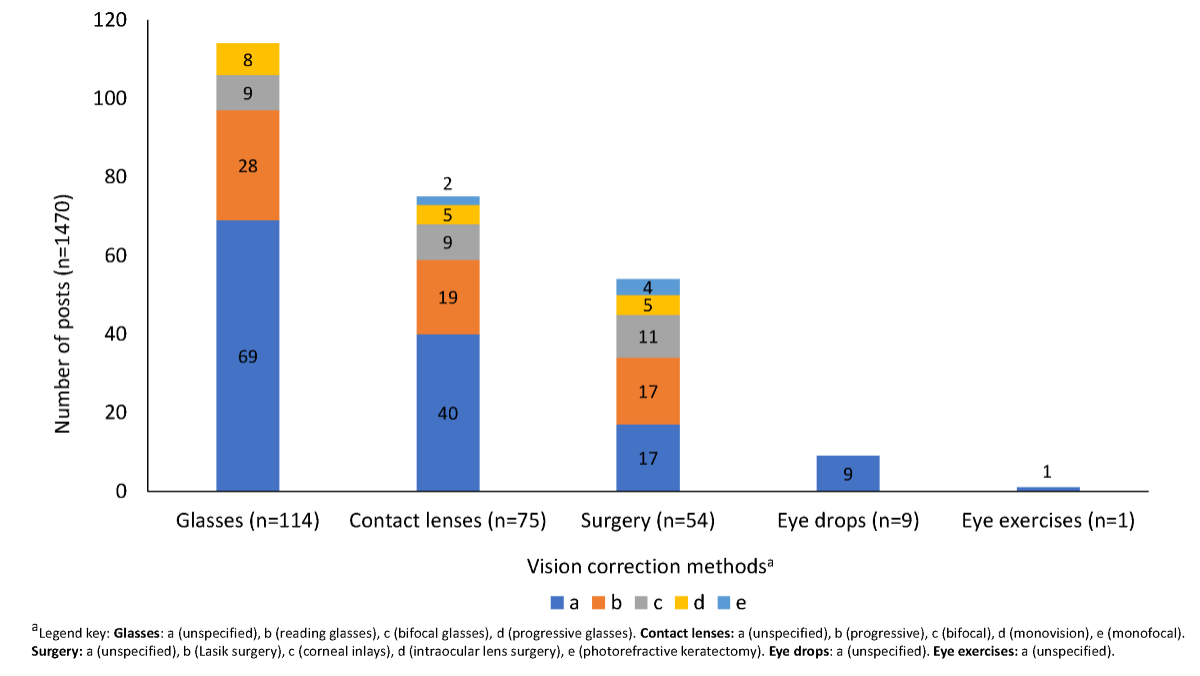
Vision correction methods reported in social media posts.

Figure 3 provides an overview of the vision correction methods reported in the social media posts. The most frequently reported vision correction options included glasses (114/1470 (7.8%)—of which 60.5% (69/114) were not specified further, 24.6% (28/114) were referred to as reading glasses, 7.9% (9/114) were bifocal glasses, and 7.0% (8/114) were progressive glasses—and contact lenses (75/1470, 5.1%)—of which 53.3% (40/75) were not specified further, 25.3% (19/75) were progressive, 12.0% (9/75) were bifocal, 6.7% (5/75) were monovision, and 2.7% (2/75) were described as monofocal. Other vision correction options discussed included surgery (54/1470, 36.7%)—which included 44.7% (17/38) posts that did not specify the type of surgery, 44.7% (17/38) posts that specified Lasik surgery, 28.9% (11/38) posts that specified corneal inlays, 13.2% (5/38) posts that specified intraocular lens surgery, 10.5% (4/38) posts that specified photorefractive keratectomy—and eye drops, as part of a clinical trial (9/1470, 0.6%), or eye exercises (1/1470, 0.1%).

I bought a pair of bifocal glasses focusing to both distances for presbyopia.

A number of impacts were found to be associated with current vision correction options. Impacts associated with wearing glasses included feeling “fed up” with having to use reading glasses, feeling unhappy with varifocal glasses, having to remove glasses or look under them while doing close work. Impacts associated with wearing contact lenses included having to have an additional pair of glasses to be able to see middle vision.

The varifocals have worked very well for most things apart from playing the piano... a very specific middle distance when reading music, so I have another pair of glasses just for that. If I need to do very near work, like tweezing eyebrows or a manicure, I put my glasses on my head or peer from under them.

## Discussion

### Overview of Findings

The data collected in this study identified key concepts relevant to individuals with presbyopia and provided insight into how this condition is discussed online by multiple stakeholders across multiple countries. Key symptoms and vision impairment problems discussed by individuals online included difficulty reading small print, diminished ability to focus on near objects, and eye strain. Impacts of presbyopia discussed in the social media posts included difficulty reading, difficulty using electronic devices, and difficulty taking part in sport and leisure activities. Individuals with presbyopia also discussed the emotional impact of presbyopia with most expressing sadness.

### Value of Findings

The findings from this study provide qualitative insights into the lived experience of presbyopia, where there is currently limited published qualitative research. Nevertheless, the symptoms discussed by this social media population were consistent with those reported in other qualitative studies, specifically difficulties with near vision and blurred vision [5,6,8]. The findings obtained in this study also reflect those described in other qualitative studies that have explored the impact of refractive error (including individuals with presbyopia) on individuals’ daily lives [1,5-9,22]. Difficulties carrying out tasks that require near vision have also been reported in a number of other qualitative studies [5,6,8,23,24]; these include reading small print, using digital devices such as a mobile phones, self-care (for example, putting make-up on), hobbies that require detailed near vision (for example, sewing or weaving), driving, watching television, difficulties with sports and exercise, and difficulties preparing food.

Emotional impacts associated with presbyopia reported in other qualitative studies [6,8] included requiring more help from others, feeling ashamed or embarrassed, feeling scared, depressed, and isolated. Thus, while there is overlap between the published literature and the findings reported here (particularly in terms of depression and sadness), there were also differences, with shame and embarrassment not emerging from the social media review. Impacts on work have also been associated with presbyopia in the literature [6]. Thus, in general, the findings in this social media listening study corroborate other findings in the literature, arguably providing greater confidence that the findings can be generalized across populations, given the large sample size of the social media listening study, which included posts from individuals from a range of countries around the world. This study highlights the value of social media listening for identifying and confirming relevant quality of life concepts for any given disease.

The findings from this social media listening study have contributed to the development of a conceptual model of presbyopia and helped inform development of an interview guide for prospective, in-depth qualitative research and to inform the modification of a patient-reported outcome measure to assess near vision functioning in presbyopia. Knowledge of how individuals discuss their presbyopia and the key concepts associated with their condition could aid communication between health care professionals and their patients and provide a basis for a patient-reported outcome measure that could be used to evaluate quality of life. The findings add to the published evidence regarding the lived experience of presbyopia and are of value to inform the design of clinical trials and other research studies to ensure adequate measurement of the identified visual functioning and quality of life concepts.

### Study Limitations

The social media posts were analyzed in two ways—first by automated analysis and second by manual analysis. The benefit of automated analysis was that it allowed large amounts of data to be analyzed quickly and efficiently. This methodology was used to reduce the large amount of posts to a more manageable size by identifying and dismissing irrelevant posts. Using automated analysis, however, meant that some intricacies of human expression may not have been captured, and relevant posts may have been dismissed.

As the data were retrospectively collected from social media posts in the public domain, the only information about participants that could, generally, be obtained was the country of origin. Demographic or clinical information about the social media population in most instances could not be obtained; therefore, there was no way to confirm that the individuals truly had a diagnosis of presbyopia [25]. As such, it is not certain that all posts were from individuals with presbyopia (or other relevant stakeholders), and the authors acknowledge that some data may be incorrectly categorized.

When using social media as research media, certain biases need to be considered. People who post on social media represent a biased sample which may not be representative of the whole population of interest. Older people are often underrepresented on social media [26]; this needs to be considered when researching a condition such as presbyopia which becomes more prevalent with increasing age. Internet usage has increased in the older population over the last 20 years, but research shows that it is still used 20% less frequently by older populations than by younger populations and its use varies vastly based on socioeconomic factors, with lower economic status older populations being less active on the internet [27]. This study only found a small proportion of posts about quality of life from the total number of posts identified during the search. As presbyopia increases in severity with age, it is possible that individuals whose quality of life is most affected by their presbyopia are of an age where social media use is less common [26]. An alternate explanation could be that individuals with presbyopia do not discuss the impact presbyopia has on quality of life on social media. It is also acknowledged in the results that social media campaigns related to presbyopia awareness in Spain, the United States, and Japan likely led to increased tweets related to presbyopia in those countries relative to others. Thus, the data regarding the relative number of posts in each country should be interpreted with caution.

Social media listening research brings about its own ethical challenges, given individuals cannot formally consent to the use of their data. It can be argued that, since the social media posts are in the public domain, there is implicit consent that they can be read and used for research. Nevertheless, there is some guidance available regarding steps that can be taken to protect the privacy of individuals posting on social media platforms [28]. Recommendations include only collecting the data necessary to answer the research question, presenting data carefully to avoid participant identification, and understanding the risk of and not using direct text quotations from research participants. However, there is a lack of agreement on the correct way to conduct and present this form of research [29,30]. In particular, the introduction of new General Data Protection Regulation [30] guidelines in the European Union require privacy of any data by design and by default, but those guidelines do not currently offer specific solutions to ensure privacy is maintained in social media research. To ensure anonymity in this study, appropriate steps were taken to deidentify direct quotations. This included removing any identifiable information and editing quotations where necessary to ensure original quotations cannot be discovered online.

### Conclusion

Presbyopia has a substantial impact on individuals’ daily life. Individuals with presbyopia can experience particular difficulties with reading and electronic device usage that impact their daily lives and quality of life. Despite the limitations and considerations described, the social media listening methodology provides a quick and person-focused starting point to identify topics and relevant themes of interest that corroborate and provide greater ecological validity to other qualitative findings in the literature. These findings have been used to inform the design of more in-depth, prospective qualitative research and to support research outcomes. For data collected as part of a research outcomes study, the findings should be supplemented with further qualitative research (eg, interviews with individuals with presbyopia) for added rigor [31].

## Appendix

Multimedia Appendix 1 Social media search strategy.

Multimedia Appendix 2 Predefined inclusion and exclusion criteria.

Multimedia Appendix 3 Data source of relevant posts.

Multimedia Appendix 4 Country of origin of relevant posts.

## References

[1] Holden BA, Fricke Timothy R, Ho S May, Wong Reg, Schlenther Gerhard, Cronjé Sonja, Burnett Anthea, Papas Eric, Naidoo Kovin S, Frick Kevin D (2008). Global vision impairment due to uncorrected presbyopia. Arch Ophthalmol.

[2] Charman WN (2008). The eye in focus: accommodation and presbyopia. Clin Exp Optometry.

[3] Charman WN (2014). Developments in the correction of presbyopia I: spectacle and contact lenses. Ophthalmic Physiol Opt.

[4] Fricke TR, Tahhan N, Resnikoff S, Papas E, Burnett A, Ho SM, Naduvilath T, Naidoo KS (2018). Global Prevalence of Presbyopia and Vision Impairment from Uncorrected Presbyopia: Systematic Review, Meta-analysis, and Modelling. Ophthalmology.

[5] Goertz AD, Stewart WC, Burns WR, Stewart JA, Nelson LA (2014). Review of the impact of presbyopia on quality of life in the developing and developed world. Acta Ophthalmol.

[6] Kandel H, Khadka J, Goggin M, Pesudovs K (2017). Impact of refractive error on quality of life: a qualitative study. Clin. Experiment. Ophthalmol.

[7] Frick KD, Joy SM, Wilson DA, Naidoo KS, Holden BA (2015). The Global Burden of Potential Productivity Loss from Uncorrected Presbyopia. Ophthalmology.

[8] Lu Q, Congdon N, He X, Murthy GVS, Yang A, He W (2011). Quality of life and near vision impairment due to functional presbyopia among rural Chinese adults. Invest Ophthalmol Vis Sci.

[9] (2017). What is Presbyopia?. American Academy of Opthalmology.

[10] Snelson CL (2016). Qualitative and Mixed Methods Social Media Research. International Journal of Qualitative Methods.

[11] Greenwood S, Perrin A, Duggan M (2016). Social media update 2016. Pew Research Center.

[12] Song H, Omori K, Kim J, Tenzek KE, Hawkins JM, Lin W, Kim Y, Jung J (2016). Trusting Social Media as a Source of Health Information: Online Surveys Comparing the United States, Korea, and Hong Kong. J Med Internet Res.

[13] Cook N, Mullins A, Gautam R, Medi S, Prince C, Tyagi N, Kommineni J (2019). Evaluating Patient Experiences in Dry Eye Disease Through Social Media Listening Research. Ophthalmol Ther.

[14] Gutzwiller F, Gruenberger J, Kaur V, Pathak P, Mudumby A, Cook N (2018). Generating patient insights in chronic obstructive pulmonary disease (COPD) with social media listening study. Eur Respiratory Soc.

[15] Cook NS, Kostikas K, Gruenberger J, Shah B, Pathak P, Kaur VP, Mudumby A, Sharma R, Gutzwiller FS (2019). Patients' perspectives on COPD: findings from a social media listening study. ERJ Open Res.

[16] Gabriel S, Phillips K, Slater M, Zhou J, Pathak A, Lee L (2017). The emotional journey among caregivers of patients with leukemia: The caregivers’ perspective using social media listening. J Clin Oncol.

[17] Anderson L, Bell HG, Gilbert M, Davidson JE, Winter C, Barratt MJ, Win B, Painter JL, Menone C, Sayegh J, Dasgupta N (2017). Using Social Listening Data to Monitor Misuse and Nonmedical Use of Bupropion: A Content Analysis. JMIR Public Health Surveill.

[18] Humphrey L, Willgoss T, Trigg A, Meysner S, Kane M, Dickinson S, Kitchen H (2017). A comparison of three methods to generate a conceptual understanding of a disease based on the patients' perspective. J Patient Rep Outcomes.

[19] Sjödahl Hammarlund C, Nilsson MH, Hagell P (2011). Measuring outcomes in Parkinson’s disease: a multi-perspective concept mapping study. Qual Life Res.

[20] (2019). Sales Force Social Studio. Sales Force Studio.

[21] Mohammad SM, Turney PD (2012). Crowdsourcing a word-emotion association lexicon. Computational Intelligence.

[22] Kandel H, Khadka J, Shrestha MK, Sharma S, Neupane Kandel S, Dhungana P, Pradhan K, Nepal BP, Thapa S, Pesudovs K (2017). Uncorrected and corrected refractive error experiences of Nepalese adults: a qualitative study. Ophthalmic Epidemiology.

[23] McDonnell PJ (2003). Associations of Presbyopia With Vision-Targeted Health-Related Quality of Life. Arch Ophthalmol.

[24] Williams S, Brian G, Toit RD (2012). Measuring Vision-specific Quality of Life among Adults in Fiji. Ophthalmic Epidemiology.

[25] Cesare N, Grant C, Nsoesie E (2017). Detection of user demographics on social media: A review of methods and recommendations for best practices. arXiv preprint arXiv.

[26] Schwartz HA, Ungar LH (2015). Data-Driven Content Analysis of Social Media. The Annals of the American Academy of Political and Social Science.

[27] Hunsaker A, Hargittai E (2018). A review of Internet use among older adults. New Media & Society.

[28] Moreno MA, Goniu N, Moreno PS, Diekema D (2013). Ethics of Social Media Research: Common Concerns and Practical Considerations. Cyberpsychology, Behavior, and Social Networking.

[29] Golder S, Ahmed S, Norman G, Booth A (2017). Attitudes Toward the Ethics of Research Using Social Media: A Systematic Review. J Med Internet Res.

[30] Kotsios A, Magnani M, Vega D, Rossi L, Shklovski I (2019). An Analysis of the Consequences of the General Data Protection Regulation on Social Network Research. Trans. Soc. Comput.

[31] (2009). Guidance for industry: patient-reported outcome measures: use in medical product development to support labeling claims. US Food and Drug Administration.

