# Supplementary material for: Social Media Listening to Understand the Lived Experience of Presbyopia: Systematic Search and Content Analysis Study
Source: J Med Internet Res. 2020 Sep 21;22(9):e18306. doi: 10.2196/18306 (PMC7536603; doi:10.2196/18306)
Supplement: Multimedia Appendix 1 [file jmir_v22i9e18306_app1.pdf]

Multimedia Appendix

Table 1. Social media search strategy

Domain	Search terms used
**Generic presbyopia terms**	(Presbyopia OR #presbyopia OR Long Sightedness OR Long-sightedness OR Loss of elasticity) AND (Aging eye condition OR Hardening of natural lens OR Hardened natural lens OR Hardened lens OR Far and near vision OR clear vision
**Symptom terms**	Blurring of near objects OR Near objects blurring OR Headaches OR Headache OR Eye strain OR Eyestrain OR Comprehensive eye OR Inside of eye OR Inside of the eye OR Dilating Pupils OR Low light
**Impact terms**	Objects up close OR Holding objects OR Arm's distance OR Arm length OR Difficulty in reading OR Reading OR Productivity OR Work Loss OR Driving OR Computer Usage OR Smartphone usage OR Daily Activity OR Recognizing peoples' faces OR Absence OR Office
**Lived experience terms**	OR Patient Journey OR Experience OR Patient concerns
**Vision correction terms**	OR Monovision OR Monofocal Eye Glasses OR Multifocal OR Monofocal contact lens OR Multifocal contact lens OR LASIK OR Intra ocular lens OR IOL OR Corneal Inlays OR no curative OR long lasting OR desired OR not satisfactory OR corneas reshaped OR Reshaping Corneas OR optometry care OR Corrective Lens OR Vision Correction OR Varifocal OR Bifocal OR focusing loss OR INTRACOR OR Laser blended vision OR Cornealinlays OR #CornealInlays OR Middle age OR Old Age OR elderly)

This is a Multimedia Appendix to a full manuscript published in the J Med Internet Res. For full copyright and citation information see http://dx.doi.org/10.2196/jmir.18306
