# Supplementary material for: Social Media Listening to Understand the Lived Experience of Presbyopia: Systematic Search and Content Analysis Study
Source: J Med Internet Res. 2020 Sep 21;22(9):e18306. doi: 10.2196/18306 (PMC7536603; doi:10.2196/18306)
Supplement: Multimedia Appendix 2 [file jmir_v22i9e18306_app2.pdf]

Multimedia Appendix

Table 1. Pre-defined inclusion/exclusion criteria

	**Criteria**
**Inclusion criteria**	Posts were included in the final analysis if they:<br>• Contained information about presbyopia, specifically: the symptoms experienced, diagnosis, treatment, management and HRQoL
**Exclusion criteria**	Posts were excluded from the final analysis if they:<br>• Were not related to presbyopia (e.g., posts related to other visual conditions)<br>• Contained market reports or buy/sell content<br>• Contained animal content<br>• Contained the word 'presbyopia' in common parlance but was used out of context and did not refer to the condition

This is a Multimedia Appendix to a full manuscript published in the J Med Internet Res. For full copyright and citation information see http://dx.doi.org/10.2196/jmir.18306
