# Supplementary material for: Social Media Listening to Understand the Lived Experience of Presbyopia: Systematic Search and Content Analysis Study
Source: J Med Internet Res. 2020 Sep 21;22(9):e18306. doi: 10.2196/18306 (PMC7536603; doi:10.2196/18306)
Supplement: Multimedia Appendix 3 [file jmir_v22i9e18306_app3.pdf]

Multimedia Appendix

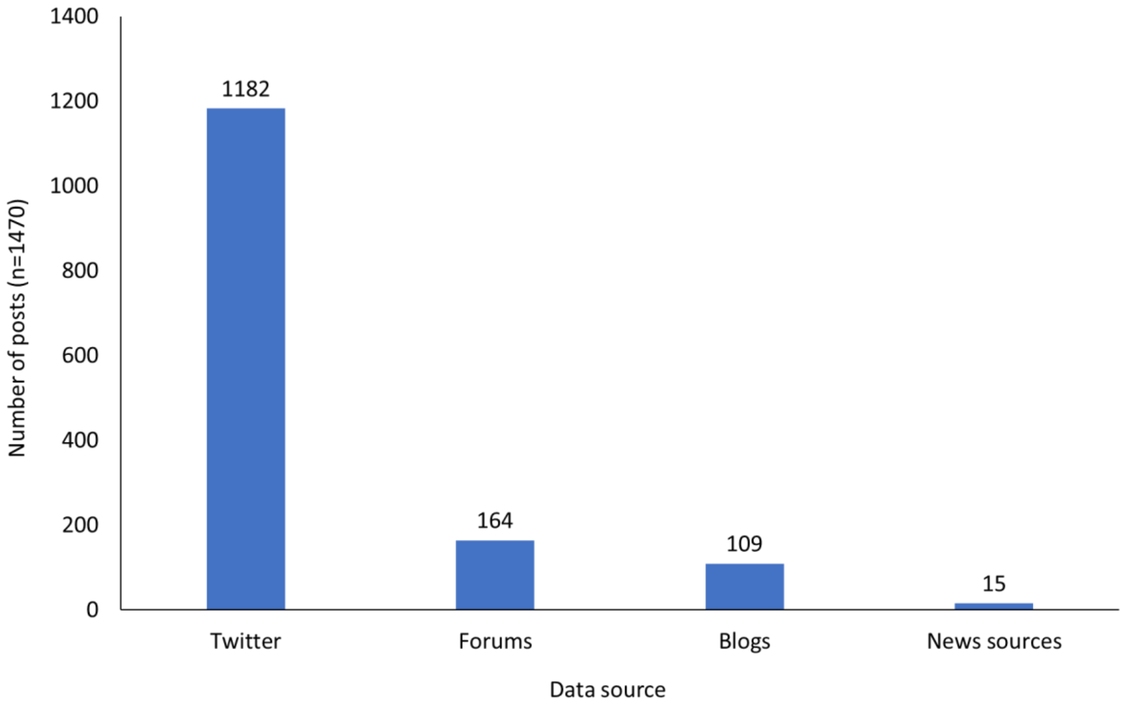

Figure 1. Data source of relevant posts

This is a Multimedia Appendix to a full manuscript published in the J Med Internet Res. For full copyright and citation information see http://dx.doi.org/10.2196/jmir.18306
